# Immune response variation in mild and severe COVID-19 patients

**DOI:** 10.5339/qmj.2024.11

**Published:** 2024-02-29

**Authors:** Samuel Stroz, Piotr Kosiorek, Edyta Zbroch, Bozena Mikoluc, Anna Stasiak-Barmuta

**Affiliations:** 1 Department of Clinical Immunology, Medical University of Bialystok, Bialystok, Poland Email: samuelstroz92@gmail.com; 2 Department of Internal Medicine and Hypertension, Medical University of Bialystok, Bialystok, Poland; 3 Department of Pediatrics, Rheumatology, Immunology and Metabolic Bone Diseases, Medical University of Bialystok, Bialystok, Poland

**Keywords:** cytometry, T-cells, vaccination, post-COVID syndrome, immunology

## Abstract

Sixty patients with COVID-19 infection were categorized into mild and severe groups, and their immune response was analyzed using flow cytometry and complete blood count. An observed increase in immune activation parameters, notably a higher percentage of CD4 lymphocytes co-expressing CD69 and CD25 molecules, and enhanced activity of the macrophage-monocyte cell line was noted in the mild group. Although Group 2 (severe COVID) had fewer CD4 cells, significant migration and proliferation were evident, with increased CD4CD69, CD8 HLA-DR+, and CD8CD69 lymphocytes. The CD4 to CD8 ratio in Group 1 suggested potential autoimmune reactions, while Group 2 indicated potential immunosuppression from severe infection and employing immunosuppressive drugs. Additionally, Group 2 exhibited an increased neutrophil count, hinting at possible bacterial co-infection. Group 1 showed differences in CD4RO and CD8RA lymphocyte populations, implying that cellular immunity plays a role in developing efficient postinfectious immunity. This intimation suggests that vaccination might mitigate the severity of the coronavirus infection and prevent complications, including long-term COVID-19.

## Introduction

Severe acute respiratory syndrome caused by coronavirus was first detected in Wuhan, China, in December 2019. In March 2020, the World Health Organization (WHO) declared the 2019 coronavirus infection a pandemic, which caused an overload of the healthcare system in most countries globally and led to substantial economic losses.^1^ According to the WHO dashboard (data observed on August 17, 2023), there are almost 770 million confirmed cases of COVID-19 and about 7 million deaths.^2^ In addition to the severe course of the infectious disease with a high mortality rate, Taleghani and Taghipour noted that many patients with COVID-19 are asymptomatic or have only mild symptoms of the disease while maintaining high contagiousness, which in turn leads to difficulties with screening, prevention, and control of the epidemic.^3^

The immune response associated with SARS-CoV-2 infection is central to the pathogenesis of COVID-19, involving hyperactivation of the innate immune response, formation of extracellular traps of neutrophils, and lymphocytopenia.^4,5^ Not all immune responses are protective, as antibody-dependent enhancement of humoral immunity may contribute to SARS-CoV-2 infection. In contrast, a T-lymphocyte response in a cell-mediated reaction may contribute to a cytokine storm. Chen et al. focussed on the fact that the immune hypothesis of the pathogenesis of coronavirus infection explains the high vulnerability of elderly patients.^6^ Because COVID-19 is pathophysiologically associated with the development of a cytokine storm, it leads to endothelial dysfunction and endotheliitis, which in turn causes the development of microvascular thrombi, ischemia, and multiple organ failure, which determines the multisystem nature of the lesion. Anka et al. also pointed out that individuals with a severe course of COVID-19 typically have eosinopenia and lymphopenia with a marked decrease in the frequency of clusters of differentiation CD4+ and CD8+ T-cells, basophils cells, and natural killers (NK) cells.^7^ Additionally, several studies have provided growing evidence that COVID-19 can cause an immune system to become dysregulated and result in the emergence of autoimmune disorders (especially vasculitis and arthritis, less often – idiopathic inflammatory myopathies, systemic lupus erythematosus and sarcoidosis, systemic scleroderma, and others).^8,9^

Therefore, identifying the immunopathological effects of COVID-19 may become a potential immunotherapy target and is essential for choosing an appropriate treatment regimen. In addition to standard supportive care, therapeutic approaches to treating COVID-19 involve immunomodulators to suppress the immune system and prevent a cytokine storm and antiviral agents to disrupt the coronavirus life cycle.^10,11^ Vaccines also play an essential role in influencing the state of the immune system. Zawilska et al. showed a significant reduction in the risk of infection and hospitalization associated with COVID-19 after vaccination.^12^

Immunosuppression may present drawbacks and benefits in severe COVID-19 patients with suspected bacterial co-infections. While dampening harmful inflammation, immunosuppression could also hamper bacterial clearance by inhibiting neutrophil and T-cell responses. However, unchecked inflammation exacerbates tissue damage. Therefore, judicious immunotherapy balancing sufficient antimicrobial immunity with controlled inflammation may be optimal. Further research is required to fully define the complex interplay between immunosuppression, secondary infections, and hyperinflammation in severe COVID-19 cases. Careful modulation of the immune response is needed to curb inflammation while preserving bacterial killing capacity.

According to Guziejko et al., symptoms of COVID that persist for longer than three months are the predominant criteria used to define the term ”post-COVID-19 condition” (PCC).^13^ This complication may include pulmonary, cardiovascular, neurological, renal, hematological, gastrointestinal, endocrine, and psychosocial symptoms that develop due to immunological impairments.^14,15^

In light of the prediscussed data, the goal of this project is to assess the influence of SARS-CoV-2 viral infection on a few immune response measures in patients with mild or severe COVID-19 and to identify the differences in the disease’s progression in terms of PCC.

## Materials and Methods

### Study design and participants

This prospective cohort study was conducted over an asynchronous time spanning from March 9, 2021, to December 28, 2021. Sixty patients with PCR-confirmed COVID-19 infection were categorized into two groups based on the symptom severity: mild without hospitalization and long COVID syndrome (21 patients) and severe (39 patients), requiring hospitalization and oxygen therapy for over ten days. Sample size determination was based on prior research data on immune responses in COVID-19 patients. Using a power of 0.8, an alpha level of 0.05, and an effect size derived from preliminary data, a sample size of 60 was deemed sufficient to detect statistically significant differences between the mild and severe groups. G*Power software was utilized for these calculations. The Medical University of Bialystok Institutional Review Board approved the study on February 21, 2021 (No 1046-A).

### Treatment and sample collection

Patients were administered standard treatment, which included dexamethasone and anticoagulant prophylaxis. Blood samples were collected from the severe group between the tenth and the fourteenth day of hospitalization. Blood samples were obtained for patients in the mild group through scheduled visits to outpatient clinics associated with the research institute. Specifically, 10 to 14 days post-positive PCR confirmation, patients were invited to the clinics for sample collection, ensuring parity in the sampling timeline between both groups. Samples were stored in ethylenediaminetetraacetic acid-laden tubes.

### Laboratory analysis

Blood samples underwent staining by mixing 10 mL of monoclonal antibodies (Beckman Coulter, USA) with 100 mL of whole blood. These antibodies were pre-bound to fluorochromes. After a 20-minute incubation, samples were processed using the Coulter rapid no-wash whole blood lysis station. Analysis was conducted using the Beckman Coulter Fc 500 MCL flow cytometry analyzer, examining a minimum of 10^5^ cells from each sample (Table 1).

### Statistical analysis

Before performing the Welch t-test, data was analyzed using the Shapiro-Wilk test for normality. Results from this test indicated that the data deviated from a normal distribution, prompting the exploration of alternative testing methods. The Welch t-test was chosen due to its robustness in handling unequal variances between groups. Additionally, Levene’s test for equality of variances was conducted and indicated that the two groups had unequal variances, further justifying the use of the Welch t-test. All statistical analyses were executed using the SPSS software, version 27.

## Results

Several immunological differences emerged during the comparative analysis of the groups under study (Table 2). In Group 1, CD3, NK cells, and T lymphocytes (CD3) were prevalent when labeled with CD62L, CD4, and CD4HLA lymphocytes. In Group 2, there was a higher presence of CD4CD69, CD8 (including those positive for HLA-DR), CD8CD69 lymphocytes, CD4CD25 cell counts, and a noticeable difference in CD4RO and CD8RA lymphocyte subsets.

Differences were also observed in eosinophil, basophil, and monocyte counts in standard blood examination, with a significant variation in peripheral blood lymphocyte count. Additionally, in the lymphocyte formula, the number of neutrophils was higher in Group 2, with a trend towards lymphopenia compared to Group 1. However, basophils, eosinophils, erythrocytes, hemoglobin, and platelet levels remained within the reference values for most patients in both groups.

Severe COVID-19 patients, especially those not vaccinated, may benefit more from vaccination during recovery than mild cases. The study findings showed severe COVID-19 is associated with signs of immunosuppression like reduced CD4+ T-cells and CD4/CD8 ratios. This effect could impede the development of robust postinfectious immunity. However, vaccination can provide targeted stimulation to Train the immune system and promote durable T-cell memory against SARS-CoV-2. While mild cases exhibited markers indicating the natural evolution of immunity post-infection, severe cases displayed chronic inflammation and compromised immunity. Therefore, timely vaccination for unvaccinated severe COVID-19 patients could prove advantageous by priming T and B cell responses and overcoming potential immune dysfunction. Maximum vaccine effectiveness may be achieved by optimizing timing based on the recovery stage. Vaccination merits strong consideration for unvaccinated severe COVID-19 patients to boost immune protection that their immunosuppressed state may otherwise hinder.

## Discussion

The observations from Group 1 suggest a transmembrane signal of viral infiltration. The presence of HLA-positive CD4 lymphocytes indicates prolonged circulation in the bloodstream, suggesting an activated state of cells even post-COVID-19. Conversely, Group 2 exhibits markers indicating active migration and proliferation of CD4 cells, increased proinflammatory cytokine production, and the onset of a chronic inflammatory response. The differing CD4/CD8 ratios between the two groups suggest varying immune responses, potentially pointing to a hyperimmune reaction or a leaning toward immunosuppression.

The difference in the CD4RO and CD8RA lymphocyte subsets highlights the development of efficient postinfectious cellular immunity, especially in Group 1. Despite an increased humoral response, a decline in cellular immunity in Group 2 can suggest the onset of autoimmune processes. The enhanced prevalence of the CD14/80 marker in patients from Group 2 indicates an active phase of antigen presentation, denoting the body’s increased efforts against the pathogen. The increase in CD4CD25 cells in Group 2 shows a heightened number of regulatory T-cells that could play a role in immunosuppression.

The discrepancies in immune response markers, such as the eosinophil and basophil counts, and the significant difference in the total lymphocyte count, emphasize the chronic inflammatory response in patients with severe infections. The elevated number of neutrophils in Group 2 might suggest a potential bacterial infection, coupled with the state of immunosuppression.^16^ The lymphopenia trend in Group 2 can be a natural response to viral invasion and could also predict future complications.

The observed immunological responses and the continued use of immunosuppressive therapeutic regimens highlight their importance in counteracting excessive immune system activation. In individuals not requiring hospitalization, specific lymphocytes suggest the development of strong postinfectious immunity, vital for herd immunity. Vaccination remains crucial in reducing COVID-19 severity, protecting against primary symptoms, and potentially safeguarding against PCC effects.

The dominance of HLA-positive CD4 lymphocytes in Group 1 aligns with prior studies,^17,18^ suggesting prolonged circulation in the bloodstream, indicative of a sustained activated cellular state post-COVID-19. This potentially enhanced cellular immune response echoes findings from contemporary research into post-viral immune responses.^19^

In Group 2, the prevalence of markers such as CD4CD69 and CD8CD69 has been previously linked with heightened proinflammatory cytokine production. This suggests a chronic inflammatory response, a trend observed in patients experiencing more severe COVID-19 progression.^18,20^ Variations in the CD4/CD8 ratio between the two groups have been a topic of continued research, with prior findings highlighting similar tendencies towards either hyperimmune reactions or immunosuppression.

In understanding the multifaceted immune responses to SARS-CoV-2 infection, Toor et al. explored the role of T-cells in patients diagnosed with COVID-19.^17^ They documented the emergence of coronavirus-specific CD4+ and CD8+ T-cells in peripheral blood within the initial fortnight post-symptom onset. Predominantly, CD4+ T-cells displayed a central memory phenotype, characterized by elevated cytokine production, while CD8+ T-cells demonstrated an effector phenotype with pronounced expression of perforin and granzyme basophils. Furthermore, memory CD4+ and CD8+ T-cells expressing CD38 and HLA-DR were elevated in cases of severe disease relative to their asymptomatic counterparts. Concomitantly, regulatory T-cell levels in patients with severe COVID-19 were diminished. This led the investigators to postulate that anomalies within T-cell subsets might potentiate severe inflammatory afflictions and even COVID-19 recurrence.

Zheng et al. highlighted that individuals grappling with severe COVID-19 presented elevated CD4+ T-cell levels but diminished secretion of interferon-γ, interleukin-2, and tumor necrosis factor-α compared to both healthy individuals and moderate case counterparts.^21^ Additionally, these patients exhibited increased levels of CD8+ T-cells expressing HLA-DR and T-cell immunoreceptors with Ig and ITIM domains. This mirrors the immunological patterns observed in certain chronic infections where CD4+ T-cell function is compromised, leading to overactivation and potential exhaustion of CD8+ T-cells, which might undermine antiviral immunity. Interleukins, as regulators of immune cell activity, emerged as significant markers for subsequent investigations.

Song et al. discerned no appreciable disparities in absolute counts of leukocytes, neutrophils, and platelets when contrasting mild and severe COVID-19 cases.^22^ However, patients with severe disease had considerably diminished counts of lymphocytes, CD3+ T-cells, CD4+ T-cells, CD8+ T-cells, and NK-cells. Concurrently, basophil counts were elevated, mirroring the results of the current comparative analysis except for neutrophil counts. Hallek et al. proposed that PCC, afflicting around 15% of unvaccinated adults infected with SARS-CoV-2, is likely an upshot of chronic inflammation, autoimmune reactions, endothelial damage, and persistent viral presence.^23^

Severe COVID-19 is linked with lymphopenia, instigating hyperinflammation, given that lymphocytes play a pivotal role in resolving postinfectious inflammation, as discussed by Yong.^24^ Lymphopenia and elevated proinflammatory neutrophils emerge as an independent prognostic marker for COVID-19 severity, persistence, and associated mortality. Su et al. executed a meticulous analysis of immunological responses in 309 patients following a span of 2-3 months post SARS-CoV-2 infection.^25^ Those with PCC showcased an immunogram enriched in CD4+ T-cells, proinflammatory monocytes, cytotoxic effector CD8+ T-cells, NK-cells, and memory basophils. At the same time, those without PCC displayed antiinflammatory attributes in their cytograms. Sette and Crotty delineated that patients with severe COVID-19 exhibited increased circulating CD8+ T-cells and monocytes with heightened expression of proinflammatory cytokines, underpinning a potential nexus between autoimmune processes, hyperinflammation, and ensuing symptoms in PCC.^26^

An intriguing observation from Glynne et al. is that while asymptomatic individuals had diminished CD8+ T-cell counts and augmented CD28 expression on central memory cells, patients with PCC following a mild disease course showed reduced counts of both CD4+ and CD8+ effector memory T-cells. T-cell anomalies persisted for several months after a mild disease course.^27^

Comparative studies have shown the potential to investigate the immunological status across various groups of COVID-19 patients, including those with differing disease severities and even asymptomatic or healthy individuals.^28^ In a study conducted by Wiech et al., they examined a group of patients who experienced a severe course of coronavirus infection.^29^ Their findings revealed a significant increase in the number of final effector cells CD8+ CD57+ six months after infection, accompanied by a substantial reduction in the population of naïve T-cells. Moreover, there was an elevated production of granzyme in basophils and interferon-gamma. Conversely, patients with a mild course exhibited an increase in naïve T-cells and a decrease in CD4+ regulatory T-cells.

Interestingly, the study suggested that individuals from all severity groups might be susceptible to long-term COVID-19 symptoms. Importantly, astheno-cognitive impairment was not necessarily attributed to T-cell dysfunctions, nor was unresolved inflammation predominantly observed in the severe group. Thus, it underscores the significance of categorizing patients based on their post-COVID symptoms.

Similarly, Sekine et al. conducted an analysis that demonstrated lower CD4+ and CD8+ T-cells in patients with moderate and severe COVID-19 compared to a control group.^30^ However, they observed an increased expression of activation markers such as CD38, CD69, Ki-67, and programmed cell death protein 1 (PD-1) on CD4+ T-cells and CD38, CD39, CD69, cytotoxic T-lymphocyte-associated protein 4 (CTLA-4), HLA-DR, Ki-67, lymphocyte-activation gene 3 (LAG-3), and T-cell immunoglobulin 3 (TIM-3) on CD8+ T-cells. This heightened activity of CD38 expression was associated with a robust inflammatory environment in severe cases, consistent with previous findings. Notably, the study incorporated numerous other expression markers, warranting further exploration to understand T-cell activation and regulatory mechanisms comprehensively.

Moreover, Hermens and Kesmir observed lymphopenia and a reduction in the numbers of CD4 and CD8 T-cells in COVID-19 patients, with severity correlating with the extent of these reductions.^31^ They also noted an increased proportion of activated CD38+ and HLA-DR+ cells in severe COVID-19 cases, suggesting a triad of factors: overactivation, exhaustion, and susceptibility to apoptosis.

Peluso et al. reported that patients requiring hospitalization or intensive care during the acute phase of infection exhibited heightened CD4+ T-cell reactivity during recovery.^32^ However, four months later, these levels aligned with patients with a mild course. Similarly, increased CD8+ T-cell activity was observed, particularly in cases with pre-existing respiratory system diseases during hospitalization. These findings indicate the interplay of various clinical factors affecting distinct components of the immune response, underscoring the importance of carefully considering these factors when defining study cohorts.

The study comprehensively examines a broad spectrum of immune response markers, offering a detailed view of the post-COVID-19 immune landscape. The distinct differentiation between patient groups based on disease severity further refines the insights obtained. The findings present an opportunity for further research into the long-term impacts of COVID-19 and potential therapeutic implications.

## Limitations

1. Potential variability in individual immune responses may be overlooked in group-based analyses.
2. Lack of extended follow-up data limits understanding of the long-term persistence of immunological distinctions.
3. External factors, such as concurrent infections or individual health conditions, might influence observed immune reactions.

## Conclusions

In a cohort of patients recovering from severe COVID-19, increased markers indicating immune system activation were noted, particularly in antigen-presenting cells co-expressing CD80. Additionally, an elevated proportion of activated CD4 lymphocytes co-expressing CD69 and CD25 was observed, signifying a robust immunological reaction and proliferation during PCC. Conversely, individuals recovering from COVID-19 who did not require hospitalization demonstrated a higher proportion of CD4CD45RO and CD4CD62L lymphocytes, suggesting the evolution of potent postinfectious immunity, an integral component of herd immunity. The first group’s augmented CD4/CD8 ratio may indicate a predisposition toward autoimmune events. For those with a severe infection trajectory, a diminished ratio could suggest a tilt towards immunosuppression, likely due to the intense infection and the use of immunosuppressive medications, such as dexamethasone.

Notwithstanding, immunosuppressive treatments, encompassing corticosteroids, are presently deemed the most efficacious, given the hyperactivation of the immune system and the propensity to trigger autoimmune disorders.

Delving deeper into the nuances of the immune response to SARS-CoV-2 infection can pave the way for novel therapeutic strategies targeting the overarching inflammatory reaction stemming from a cytokine storm’s onset. Comprehensive knowledge of the humoral response elicited by vaccinations promises to optimize preventive strategies against potential viral outbreaks in the future. This is particularly relevant when considering the formulation and dosing for individuals under therapeutic immunosuppression or those experiencing the natural physiological aging process.

Future investigations could focus on understanding T-cells’ regulatory cytokine expression, comparing the immunological response in asymptomatic individuals, and longitudinally monitoring patients with designated control checkpoints.

## Patient Consent

Informed consent was obtained from all individuals included in this study.

## Statement of Ethics

The Institutional Review Board provided ethical approval for this study at the Medical University of Bialystok.

## Conflicts of Interest

The authors have no conflicts of interest to declare.

## Data Availability

The authors confirm that the data supporting the findings of this study are available in the article.

## Author Contributions

SS: Mentor, Study concept and design, Data Analysis, Manuscript writing, and Manuscript editing. PK: Data collection, Data analysis, Literature review, Manuscript writing, and Manuscript editing. EZ: Data collection, Data analysis, Literature review, Manuscript writing, and Manuscript editing. BM: Data analysis, Literature review, Manuscript writing, and Manuscript editing. ASB: Literature review, Manuscript writing, and Manuscript editing.

## Figures and Tables

**Table 1.** Monoclonal antibodies used for staining T-, basophils-, NK-, natural killer T-cells, regulatory T-cells, and monocytes.

The type of cell	T-cells	NK cells	Natural killer T-cells	B cells	Regulatory T-cells	Monocytes
Fluorochrome	CD3-PC5, CD4-PC5, CD8-PC5, HLADR-FITC, CD69-PE, CD45RO-PE, CD45RA-FITC, CD62-FITC, and CD38-PE	CD16-PE, CD56-PE, and CD62-FITC	CD3-PC5, CD16-PE, and CD56-PE	CD19-PC5	CD4-PC5, CD25-FITC, and CD127-PE	CD14-PE, CD80-FITC, and CD86-PE

Source: Created by the authors.

**Table 2.** Comprehensive analysis of lymphocyte differentiation clusters and general blood analysis indicators across two groups.

Parameters	Group 1, M cell/ml	Group 2, M cell/ml	T	p
Lymphocytes differentiation clusters 4, 16, 56, and 8				
CD3	72.18	69.32	0.97	0.33
NK	18.16	16.83	0.56	0.57
Natural killer T-cells	4.20	4.45	0.21	0.83
CD4	46.13	38.87	2.06	0.04
CD4HLA	13.21	17.7	2.36	0.02
CD4CD69	1.81	4.15	−2.90	0.005
CD8	22.59	30.44	−2.25	0.029
CD8HLA	17.02	27.79	−3.45	0.001
CD8 CD69	7.23	13.33	−3.49	0.0009
Lymphocytes differentiation clusters 45 RA/ RO, 19, 14, and 80				
CD4RA	30.05	30.88	−0.19	0.848
CD4RO	62.7	51.02	2.48	0.016
CD8RA	57.86	51.25	1.605	0.115
CD8RO	30.97	32.09	−0.289	0.77
CD19	6.67	14.9	−3.13	0.003
NK TLR4	3.25	4.5	−1.202	0.23
CD3 CD62L	54.38	43.9	2.146	0.036
NK CD62L	26.91	27.23	−0.108	0.91
CD14 CD80	10.46	19.94	−3.4	0.002
Lymphocytes differentiation clusters 25, 86, and 127				
CD14 CD86	74.89	70.08	0.67	0.504
CD4 CD25	7.4	10.45	−2.519	0.0148
CD4 CD25H	3.34	3.61	−0.38	0.7
CD4 CD25 CD127	30.97	32.09	−0.28	0.77
CD4/CD8	8.41	1.71	1.26	0.219
General blood analysis indicators				
Lymphocytes, n*10^12^	6.29	9.83	−2.49	0.016
Lymphocytes, n%	29.5	18.6	3.53	0.0008
Neutrophils, n*10^12^	3.52	7.67	−3.11	0.0034
Neutrophils, n%	57.228	69.81	−3.48	0.0009
Monocytes, n*10^12^	0.59	0.6	−0.076	0.93
Monocytes, n%	9.58	7.86	1.9	0.061
Eosinophils, n*10^12^	0.15	0.1	0.92	0.36
Eosinophils, n%	2.67	1.57	1.049	0.3
Basophiles, n*10^12^	0.033	0.032	0.26	0.789
Basophiles, n%	0.57	0.42	1.55	0.127
Erythrocytes, n*10^12^	4.45	4.23	1.3	0.19
Hemoglobin, mmol/dl	13.45	13.05	0.79	0.42
Platelets, n*10^9^	241.59	211.44	1.3	0.19

Note: M – median; T – modified Student’s (Wilk’s) test.
